# DCD liver transplant in patients with a MELD over 35

**DOI:** 10.3389/fimmu.2023.1246867

**Published:** 2023-09-04

**Authors:** Raphael P. H. Meier, Miguel Nunez, Shareef M. Syed, Sandy Feng, Mehdi Tavakol, Chris E. Freise, John P. Roberts, Nancy L. Ascher, Ryutaro Hirose, Garrett R. Roll

**Affiliations:** ^1^ Division of Transplant Surgery, Department of Surgery, University of California, San Francisco, San Francisco, CA, United States; ^2^ Department of Surgery, University of Maryland School of Medicine, Baltimore, MD, United States

**Keywords:** liver transplantation, high model for end-stage liver disease score, donation after circulatory death, donation after brain death, ischemia-reperfusion, acute rejection, chronic rejection, renal failure

## Abstract

**Introduction:**

Donation after circulatory death (DCD) liver transplantation (LT) makes up well less than 1% of all LTs with a Model for End-Stage Liver Disease (MELD)≥35 in the United States. We hypothesized DCD-LT yields acceptable ischemia-reperfusion and reasonable outcomes for recipients with MELD≥35.

**Methods:**

We analyzed recipients with lab-MELD≥35 at transplant within the UCSF (n=41) and the UNOS (n=375) cohorts using multivariate Cox regression and propensity score matching.

**Results:**

In the UCSF cohort, five-year patient survival was 85% for DCD-LTs and 86% for matched-Donation after Brain Death donors-(DBD) LTs (p=0.843). Multivariate analyses showed that younger donor/recipient age and more recent transplants (2011-2021 versus 1999-2010) were associated with better survival. DCD vs. DBD graft use did not significantly impact survival (HR: 1.2, 95%CI 0.6-2.7). The transaminase peak was approximately doubled, indicating suggesting an increased ischemia-reperfusion hit. DCD-LTs had a median post-LT length of stay of 11 days, and 34% (14/41) were on dialysis at discharge versus 12 days and 22% (9/41) for DBD-LTs. 27% (11/41) DCD-LTs versus 12% (5/41) DBD-LTs developed a biliary complication (p=0.095). UNOS cohort analysis confirmed patient survival predictors, but DCD graft emerged as a risk factor (HR: 1.5, 95%CI 1.3-1.9) with five-year patient survival of 65% versus 75% for DBD-LTs (p=0.016). This difference became non-significant in a sub-analysis focusing on MELD 35-36 recipients. Analysis of MELD≥35 DCD recipients showed that donor age of <30yo independently reduced the risk of graft loss by 30% (HR, 95%CI: 0.7 (0.9-0.5), p=0.019). Retransplant status was associated with a doubled risk of adverse event (HR, 95%CI: 2.1 (1.4-3.3), p=0.001). The rejection rates at 1y were similar between DCD- and DBD-LTs, (9.3% (35/375) versus 1,541 (8.7% (1,541/17,677), respectively).

**Discussion:**

In highly selected recipient/donor pair, DCD transplantation is feasible and can achieve comparable survival to DBD transplantation. Biliary complications occurred at the expected rates. In the absence of selection, DCD-LTs outcomes remain worse than those of DBD-LTs.

## Introduction

Donation after circulatory death donor (DCD) liver transplantation (LT) remains an underutilized option in the U.S. The use of DCD liver grafts is increasing, but overall remains low (1% in 2000, 6% in 2010, and 11% in 2020) (1). Ischemia-reperfusion injury, early allograft dysfunction and their impact on renal function and biliary complications are not well tolerated by high Model for End-Stage Liver Disease (MELD) recipients (2). We (3), and others (2, 4, 5), previously designed DCD risk scores to predict outcomes, however, the corollary is that high MELD recipients are not suitable for DCD-LT. Additionally, the resulting increase in resource utilization during recovery from transplant is a concern. Consequently, patients with a high MELD are rarely transplanted with livers from DCD donors despite the paucity of outcome data after DCD-LT in patients with a high MELD. The expectation is that a Donation after Brain Death donor (DBD) will become available within a reasonable time frame. This is, unfortunately, not always the case in regions with high median MELD at transplant, and patients with a high MELD continue to experience waitlist mortality and dropout. We previously compared the different options, including DCD, DBD, and living donors (6), and it is clear that DCDs remain an important option to consider, including for high MELD recipients. Machine preservation can be used to reduce risks of DCD-LT (7), but implementation of this resource-intensive advancement has been rationed as we await real-world outcome and resource utilization data, and alternatives to machine perfusion must be considered as part of this discussion (8).

We hypothesized that select recipients with a lab MELD≥35 might tolerate increased ischemia-reperfusion and lead to acceptable morbidity/mortality, renal recovery, and biliary complications rates after DCD-LT using static cold storage. To test this hypothesis, we analyzed our local cohort of DCD-LTs in patients with MELD≥35 and compared them to matched DBD-LTs. We also compared our single-center cohort with the national DCD/DBD-LTs cohort.

## Methods

### Study design and patients

All research was conducted in accordance with both the Declarations of Helsinki and Istanbul. Approval was obtained by the Institutional Review Board of the University of California, San Francisco (UCSF; IRB 15-18341) for retrospective data review. Donor and recipient data were extracted from the UNOS database and included all consecutive adult liver transplants performed between 1989 and 2021. The study period was maximized to optimize the number of DCD-LTs in MELD≥35. Study groups included adult LT recipients (≥18yo) with a MELD≥35 at the time of transplant (lab-MELD used in the corresponding match run). Patients with exception points and those who received machine-perfused livers were excluded.

### Recipient selection and organ allocation

Recipients diagnosed with End-Stage Liver Disease were evaluated for candidacy by a multidisciplinary team and placed on the transplant waiting list (9). Donor and recipient selection and procedures were performed as previously described (3, 9, 10).

### Donor selection, procurement, and liver transplantation

Maastricht class III DCD donor selection at UCSF generally included donors younger than 60 years, with a donor warm ischemia time (WIT) ≤30 minutes (3). Intraoperative assessment of liver grafts was performed by the donor surgeon. Large droplet steatosis greater than 10-15% was avoided. Recipients undergoing retransplantation were generally not considered for DCD-LT. Procurement was performed using the “super-rapid technique” (11, 12). LT was performed as previously described (9), typically utilizing the piggyback technique. Immunosuppression typically consisted in Solumedrol induction (1gm) and maintenance with tacrolimus (for initial trough levels of 10-15 ng/ml) and Mycophenolate mofetil (1000-1500mg BID). No depleting agent were used.

### Matching methods

Matching was restricted to observations that had propensity scores in the extended common support region, which extends the common support region by 0.25 times a pooled estimate of the common standard deviation of the logit of the propensity score. Weighted matched standardized differences and variance ratios for the propensity score model covariates were used to assess sample balance after matching. Acceptable balance was defined by a maximum of 0.1 for the absolute value of standardized difference and by values within the 0.5-2 range for variance ratio. Only patients with no missing covariates were used for as close as possible to 1:1 matching. To account for the matched nature of the sample, Cox models were stratified on the matched pairs. The propensity score models included all variables that were significantly different between DCD and DBD-LTs in both cohorts.

### Statistical analysis

Continuous variables were expressed as means and standard deviations. Counts and percentages were provided for categorical variables. Comparison between groups was performed using the Student’s t-test for continuous variables and the chi-squared test for binary or categorical variables. Survival analyses were performed using the Kaplan–Meier curves, and differences were assessed using log-rank tests. Uni-/multivariate Cox proportional-hazard regression was used to compute hazard ratios (HR). Estimation methods using average of preceding and following values were used for imputing missing postoperative creatinine values. We used IBM SPSS Statistics version 26 for all computations (IBM Corp. Armonk, NY). Ninety-five percent confidence intervals (95%CI) were reported, and an exact two-sided p-value <0.05 was considered statistically significant.

## Results

### DCD utilization and MELD score in the U.S.

**Figure 1:**
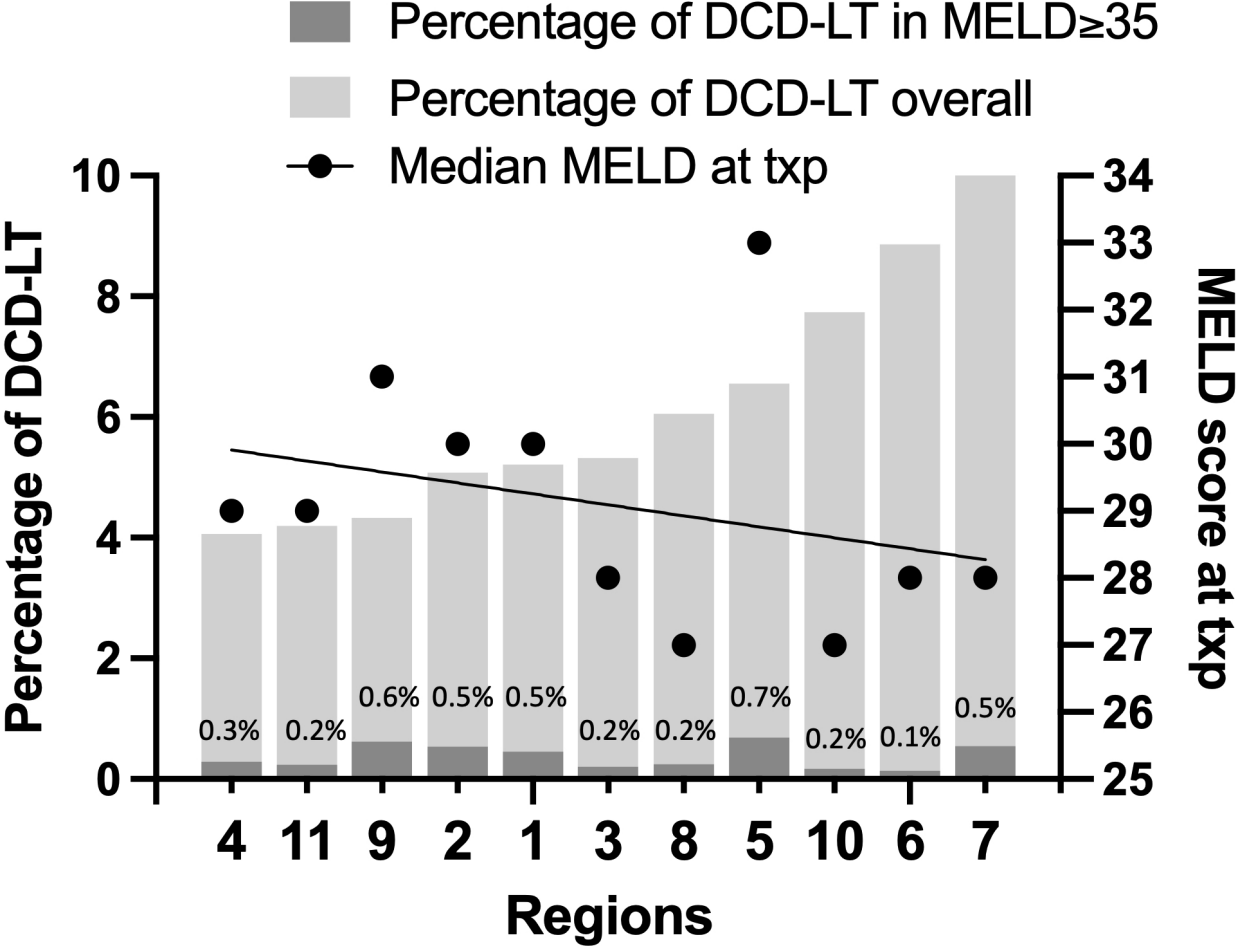
DCD graft use across UNOS regions with median MELD at transplant (as made available in 2021).

**Figure 1** displays DCD graft use across UNOS regions with corresponding median MELD at transplant (as made available in 2021). The percentage of DCD-LTs performed ranged from 4% to 10%. The percentage of those performed in recipients with a MELD≥35 ranged from 0.1% to 0.7%. There was a trend towards increased DCD use in regions with a lower MELD at transplant, with the exception of Region 5, where the median MELD at transplant was 33. Each year at UCSF (included within Region 5), an average of sixteen patients with MELD≥35 either die on the waitlist or are delisted due to being too sick for transplant (**Supplementary Figure 1**).

### Demographics of the lab MELD≥35 cohorts

The UCSF cohort included 41 DCD-LTs and 1,767 DBD-LTs performed in recipients with a transplant MELD≥35 (**Table 1**). DCD-LT recipients were older, included more patients with concomitant HCC and alcohol-related liver disease (**Supplementary Table 1**), and were more likely to be performed in the recent era (2011-2021 versus 1999-2010). The MELD at transplant was lower in DCD-LTs compared to DBD-LTs, 38.3 ± 1.8 versus 39.4 ± 1.4, p=0.001. Donor cause of death for DCD-LTs compared to DBD-LTs was more frequently anoxia (59% versus 15%) and CIT was shorter (7.8 ± 2.3 versus 9.2 ± 4.4 hours). Specific DCD variables included a donor warm ischemia time was 21.2 ± 5.2 min and average liver extraction time was 45.3 ± 19.3 min. Median follow-up was 5.1 years and 6.6 years in the UCSF DCD- and DBD-LT groups, respectively.

**Table 1:** Recipient and donor baseline characteristics of donation after circulatory death (DCD) and donation after brainstem death (DBD) liver transplantation in patients with a transplant MELD≥35 in the UCSF cohort.

Characteristics	DCD-LT with MELD≥35 (n = 41)	DBD-LT with MELD≥35 (n = 1,767)	DBD-LT with MELD≥35, matched (n = 41)	P-value^1^ (Overall)	P-value^2^ (Matched)
*Recipient factors*					
Age at transplant, years	55.4 ± 8.8	50.5 ± 10.9	55.1 ± 10.0	0.001	0.879
Gender					
- Male - Female	26 (63.4) 15 (36.6)	1,034 (58.5) 733 (41.5)	28 (68.3) 13 (31.7)	0.631	0.816
Pretransplant BMI, kg/m^2^	28.9 ± 6.6	27.1 ± 6.1	29.4 ± 6.7	0.099	0.725
MELD at transplant	38.3 ± 1.8	39.4 ± 1.4	38.1 ± 2.0	0.001	0.650
Era					
- 1989 – 2000 - 2001 – 2010 - 2011 – 2021	0 (0.0) 3 (7.3) 38 (92.7)	885 (50.1) 407 (23.0) 475 (26.9)	1 (2.4) 2 (4.9) 38 (92.7)	<0.001	0.549
*Donor factors*					
Age, years	31.1 ± 10	37.4 ± 16.2	32.9 ± 13.5	<0.001	0.500
Gender					
- Male - Female	26 (63.4) 15 (36.6)	1,054 (59.6) 713 (40.4)	31 (75.6) 10 (24.4)	0.748	0.337
BMI, kg/m^2^	25.6 ± 6.8	25.7 ± 5.6	27.5 ± 5.6	0.925	0.178
Cold ischemic time, hours	7.8 ± 2.3	9.2 ± 4.4	7.6 ± 2.4	0.001	0.738
Donor warm ischemia time, minutes	21.2 ± 4.5	NA	NA	NA	NA

Data are presented as mean ± standard deviation or n (%), unless specified otherwise.

DCD, Donation after death; DBD, donation after brainstem death; LT, liver transplantation; BMI, body mass index, EtOH, ethanol use; HBV, hepatitis B virus; HCV, hepatitis C virus; NASH, nonalcoholic steatohepatitis, PBC, primary biliary cholangitis; PSC, primary sclerosing cholangitis; A1AT, alpha-1 antitrypsin; MELD, Model For End-Stage Liver Disease; CNS, central nervous system; NA, not applicable.

^1^ DCD vs. DBD ^2^ DCD vs. matched DBD. Student t-test for continuous variables, X^2^ test for binary or categorical variables (global p-value).

The UNOS cohort included 375 DCD-LTs and 17,677 DBD-LTs (**Table 2**). DCD-LTs were significantly more likely to be performed in the recent era, had younger donor age, included more male donors, had lower donor BMI, and had shorter CIT compared to DBD-LTs. Median follow-up was 2.9 years and 3.0 years in the UNOS DCD- and DBD-LT groups, respectively. The etiology of liver disease is provided in **Supplementary Tables 2**. There were no differences in the presence of portal vein thrombosis or transjugular intrahepatic portosystemic shunt between DCD and DBDs (6% for both and in both groups).

**Table 2:** Recipient and donor baseline characteristics of donation after circulatory death (DCD) and donation after brainstem death (DBD) liver transplantation in patients with a transplant MELD≥35 in the UNOS cohort.

Characteristics	DCD-LT with MELD≥35 (n = 375)	DBD-LT with MELD≥35 (n = 17,677)	DBD-LT with MELD≥35, matched (n = 358)	P-value^1^ (Overall)	P-value^2^ (Matched)
*Recipient factors*					
Age at transplant, years	51.2 ± 11.6	51.4 ± 11.7	52.1 ± 11.8	0.815	0.329
Gender					
- Male - Female	215 (57.3) 160 (42.7)	10,812 (61.2) 6,865 (38.8)	213 (59.5) 145 (40.5)	0.134	0.600
Pretransplant BMI, kg/m^2^	28.8 ± 6.3	28.9 ± 6.4	29.5 ± 6.5	0.776	0.137
MELD at transplant	40.1 ± 4.7	40.1 ± 4.4	40.1 ± 4.1	0.871	0.795
Era					
- 1999 – 2010 - 2011 – 2021	137 (36.5) 238 (65.6)	5,928 (33.5) 11,748 (66.5)	115 (32.1) 243 (67.9)	0.473	0.214
*Donor factors*					
Age, years	30.5 ± 11.8	38.4 ± 15.1	30.1 ± 11.5	<0.001	0.570
Gender					
- Male - Female	253 (67.5) 122 (32.5)	10,716 (60.6) 6,961 (39.4)	253 (67.5) 122 (32.5)	0.008	0.215
BMI, kg/m^2^	25.6 ± 5.7	26.9 ± 5.6	25.8 ± 5.0	<0.001	0.637
Cold ischemic time, hours	6.3 ± 2.3	6.6 ± 2.8	6.6 ± 2.2	0.006	0.117
Donor warm ischemia time, minutes	18.4 ± 9.6	NA	NA	NA	NA

Data are presented as mean ± standard deviation or n (%), unless specified otherwise.

DCD, Donation after circulatory death; DBD, donation after brainstem death; LT, liver transplantation; BMI, body mass index, EtOH, ethanol use; HBV, hepatitis B virus; HCV, hepatitis C virus; NASH, nonalcoholic steatohepatitis, PBC, primary biliary cholangitis; PSC, primary sclerosing cholangitis; A1AT, alpha-1 antitrypsin; MELD, Model For End-Stage Liver Disease; CNS, central nervous system; NA, not applicable.

^1^ DCD vs. DBD ^2^ DCD vs. matched DBD. Student t-test for continuous variables, X^2^ test for binary or categorical variables (global p-value).

### Matching results

Of the 1,918 DBDs (UCSF cohort) and 17,677 DBDs (UNOS cohort) with transplant MELD≥35, the matched sample included 41 and 358 DBD-LT recipients, respectively. All previously observed statistically significant differences were successfully removed in both matched cohorts (**Tables 1**, **2**).

### Perioperative characteristics and outcomes in the UCSF DCD versus DBD matched cohort

**Figure 2:**
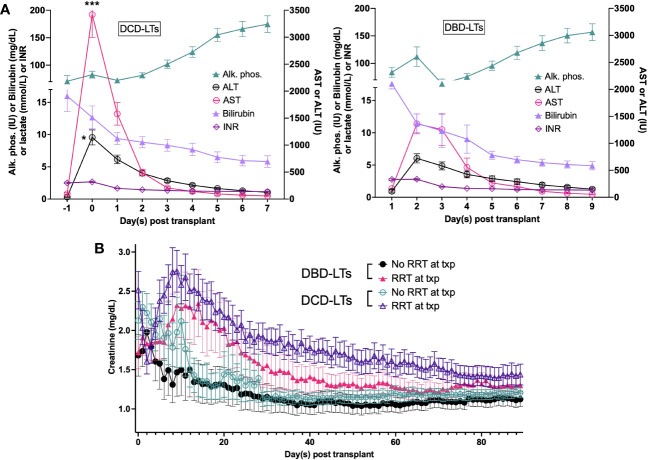
**(A)** Postoperative labs for DCD-LTs and DBD-LTs. **(B)** Postoperative creatinine values in DCD-LTs and DBD-LTs with and without renal replacement therapy (RRT) at the time of transplant (txp). p-values: DCD-LTs versus DBD-LTs, * <0.05, *** <0.0001

Of the 41 DCD-LTs vs. 41 matched DBD-LTs transplanted with a MELD≥35, 19 vs. 24 (46.3% vs. 58.5%) were in the ICU prior to transplant, more than half of the patients were receiving renal replacement therapy in both groups, and a quarter received a simultaneous kidney transplant in both groups (**Table 3**). Postoperative labs for DCD-LTs showed a two-fold increase of transaminases compared to DBD-LTs, reflecting a higher ischemia-reperfusion hit (**Figure 2**). INR, bilirubin, and alkaline phosphatase peaks were not different between the two groups. DCD-LTs had significantly higher creatinine peaks early after transplant. The median (Interquartile range (IQR)) length of stay after transplant was 11 (8–17) days for DCD-LTs and 12 (8–17) days for DBD-LTs (p=0.576). Fourteen DCD-LT recipients (34.1%) were on dialysis at discharge versus 9 (22.0%) in the DBD-LT group (p=0.219). Among the 21 patients discharged on dialysis, the median (IQR) time to renal recovery was 32 (23–61) days. Two DCD-LT recipients did not achieve renal recovery; one remained dialysis-dependent for one year, and one was an SLK who sustained DGF and died of biliary sepsis two months post-transplant. Eighteen (44%) DCD-LT recipients and fourteen (34%) DBD-LT recipients were readmitted within 90 days of transplant, on average 1.6 times in the DCD group and 1.4 times in the DBD group. Biliary complication rates were as expected for DCD- and DBD-LTs. Biliary complication (any) occurred in 11 DCD-LTs (26.8%) and five DBD-LTs (12.2%), p=0.095. Among those, five DCD-LTs developed ischemic cholangiopathy (12.2%) versus none of the DBD-LTs, p=0.021.

**Table 3:** Recipients of donation after circulatory death (DCD) versus donation after brainstem death (DBD) liver transplant recipients: perioperative characteristics and outcomes in MELD≥35 patients in the UCSF cohort.

	DCD (n=41)	DBD (n=41)	p-value*
Simultaneous liver-kidney transplant	7 (17.1)	6 (14.6)	0.762
Hospitalized in ICU pre-transplant	19 (46.3)	24 (58.5)	0.269
Renal replacement pre- transplant	24 (58.5)	23 (56.1)	0.823
Renal replacement post- transplant			
- At discharge	14 (34.1)	9 (22.0)	0.219
- At one year	1 (2.4)	0 (0)	0.314
- Duration (days) Median (IRQ)	53.3 (55.3) 33 (24-57)	53.9 (66.1) 29 (12-75)	0.983
Hospital length of stay			
- ICU after transplant (days) Median (IRQ)	5.2 (3.7) 4 (3-6)	4.4 (3.0) 4 (3-5)	0.319
- Post-transplant stay (days) Median (IRQ)	14.4 (10.5) 11 (8-17)	13.3 (7.2) 12 (8-17)	0.576
Readmissions at 90 days (n)			
- None	23 (56.1)	27 (65.9)	0.686
- One	10 (24.4)	9 (22.0)	
- Two	5 (12.2)	4 (9.8)	
- Three	3 (7.3)	1 (2.4)	
Biliary complication (any)	11 (26.8)	5 (12.2)	0.095
- Bile leak	5 (12.2)	0 (0)	0.021
- Biliary stricture	11 (26.8)	5 (12.2)	0.095
- Ischemic cholangiopathy (diffuse)	5 (12.2)	0 (0)	0.021

Data are presented as mean ± standard deviation or n (%), unless specified otherwise.

^1^ Student t-test for continuous variables, X^2^ test for binary variables.

### Survival analyses

**Figure 3:**
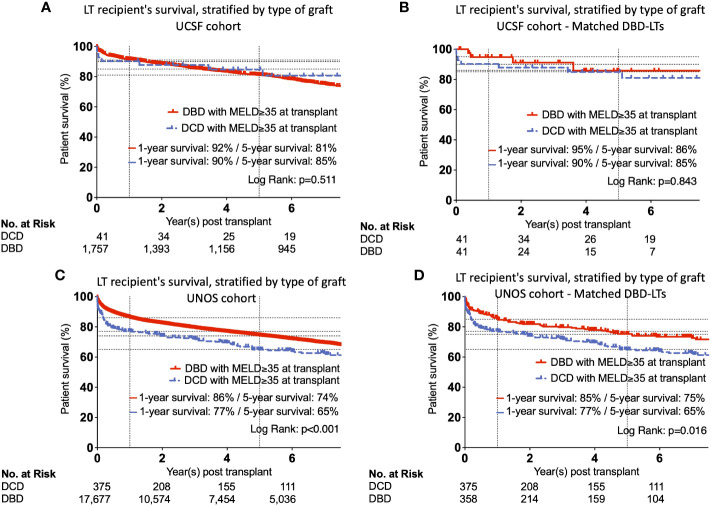
Kaplan-Meier curves showing unmatched and matched patient’s survival, stratified by type of graft in UCSF **(A, B)** and UNOS **(C, D)** cohorts.

Patient survival for all DCD- and DBD-LT recipients at UCSF with MELD≥35 during the study period did not differ (p=0.511) (**Figure 3A**). Patient survival in the matched DCD- and DBD-LT cohorts remained equivalent (p=0.843) (**Figure 3B**). The cause of death in matched groups is provided in **Supplementary Table 3**. As expected, DCD- and DBD-LTs with poor kidney function post-transplant (creatinine value >1.5, 90 days post-transplant) had a lower chance of survival compared to those with a creatinine value <1.5 at 90 days (p=0.043 and p=0.002, **Supplementary Figures 2A, B**). Graft survival analysis showed similar results with no difference between DCD- and DBD-LTs in the UCSF cohort (**Supplementary Figure 2C**) and a difference present in the UNOS cohort (**Supplementary Figure 2D**).

In the UNOS cohort, we observed lower patient survival among DCD-LT recipients at one year and five years (p<0.001) (**Figure 3C**). This difference persisted after propensity score matching of the DBD-LT group, and the difference in 1- and 5-year survival rates remained unchanged (p=0.016) (**Figure 3D**). Graft survival analysis showed similar results and differences between DCD and DBDs (**Supplementary Figure 3D**). Patient survival was well stratified by the different era, however, the differences between DCD and DBDs remained harmonious and similar to those observed in the full UNOS cohort (**Supplementary Figure 4**).

### Rejection analysis

The UCSF cohort did not show differences in terms of rejection, although the low number of occurrences did not allow a sufficiently powered statistical analysis (data not shown). The analysis of the UNOS cohort confirmed that there were no differences between DCD-LTs and DBD-LTs (unmatched and matched) in terms of acute rejection (immediate, at 6 months or 1 year) and acute or chronic rejection as a cause of graft failure (**Table 4**).

**Table 4:** Rejection episodes (immediate, at 6 months or 1 year) and as a cause of graft failure (acute or chronic rejection) for (DCD) and donation after brainstem death (DBD) liver transplantation in patients with a transplant MELD≥35 in the UNOS cohort.

Characteristics	DCD-LT with MELD≥35 (n = 375)	DBD-LT with MELD≥35 (n = 17,677)	DBD-LT with MELD≥35, matched (n = 358)	P-value^1^	P-value^2^
Rejection episode between transplant and discharge					
- Yes - No	6 (1.6) 369 (98.4)	201 (1.1) 17,476 (98.9)	3 (0.8) 355 (99.2)	0.405	0.349
Treated for rejection within 6 months					
- Yes - No	33 (8.8) 342 (91.2)	1,407 (8.0) 16,270 (92.0)	20 (5.6) 338 (94.4)	0.552	0.093
Treated for rejection within 1 year					
- Yes - No	35 (9.3) 340 (90.7)	1,541 (8.7) 16,136 (91.3)	33 (9.2) 325 (90.8)	0.676	0.957
Graft failure due to acute rejection					
- Yes - No	3 (0.8) 372 (99.2)	155 (0.9) 17,522 (99.1)	1 (0.3) 357 (99.7)	0.874	0.339
Graft failure due to chronic rejection					
- Yes - No	3 (0.8) 372 (99.2)	213 (1.2) 17,464 (98.8)	4 (1.1) 354 (98.9)	0.475	0.659

Data are presented as mean ± standard deviation or n (%), unless specified otherwise.

DCD, Donation after circulatory death; DBD, donation after brainstem death.

^1^ DCD vs. DBD ^2^ DCD vs. matched DBD. Student t-test for continuous variables, X^2^ test for binary variables.

### Multivariate analysis

We performed a Cox regression multivariate analysis including available covariates (donor/recipient age, sex, BMI, ethnicity/race, MELD, CIT, cause of liver disease, transplant era, and type of graft). In the UCSF cohort, independent factors associated with patient survival were younger donor and recipient age, shorter CIT, and recent transplant era. The type of graft used, i.e., DCD versus DBD, was not associated with the patient survival (Hazard ratio (HR) (95%CI), 1.3 (0.6-2.7), p=0.485) (**Table 5**). We performed a similar analysis in the UNOS cohort among patients with MELD≥35 and found independent predictors of improved patient survival as follows: younger donor and recipient age, recent transplant era, lower transplant MELD (i.e., closer to 35), cause of liver disease (absence of HAV, HBV, HCV, hemochromatosis, PBC, tumor or presence of PSC), Asian donor ethnicity/race, female recipient, and DBD liver graft (**Table 6**). The use of a DCD compared to a DBD graft was associated with a greater risk of death (HR (95%CI), 1.6 (1.3-1.9), p<0.001). Since the UCSF DCD-LT cohort had a lower MELD score than the UNOS cohort (38.3 ± 1.8 versus 40.1 ± 4.7), we performed a multivariate sensitivity analysis for the lower 50% of MELD≥35 recipients in the UNOS cohort. The effect of graft type was attenuated (HR 1.4 (0.9-2.1), p=0.098] (**Supplementary Table 4**).

**Table 5:** Estimated hazard ratios for liver recipient survival using a multivariate Cox proportional hazard model in the UCSF cohort (n = 1,808).

	p-value	HR^1^	95.0% CI	
			Lower	Higher
Recipient age at transplant, years	0.000	1.017	1.008	1.027
Gender, recipient, male	0.873	0.985	0.817	1.187
Recipient BMI, kg/m2	0.321	0.992	0.975	1.008
Recipient Race/Ethnicity	0.073	1.045	0.996	1.097
Etiology (recipient)	0.431	0.991	0.970	1.013
MELD	0.212	0.949	0.875	1.030
Era	0.000	1.000	1.000	1.000
Donor age, years	0.002	1.009	1.003	1.015
Gender, donor, male	0.656	1.044	0.863	1.264
Donor BMI, kg/m2	0.537	1.006	0.988	1.023
Donor Race/Ethnicity	0.601	1.009	0.975	1.044
CIT	0.023	1.022	1.003	1.041
DCD graft	0.485	1.295	0.627	2.675

^1^Multivariate Cox regression model.

DCD, Donation after circulatory death; DBD, donation after brainstem death; LT, liver transplantation; BMI, body mass index, MELD, Model For End-Stage Liver Disease; CI, confidence interval; HR, hazard ratio.

**Table 6:** Estimated hazard ratios for liver recipient survival using a multivariate Cox proportional hazard model in the UNOS cohort (n = 17,677).

	p-value	HR^1^	95.0% CI	
			Lower	Higher
Recipient age at transplant, years	0.000	1.017	1.014	1.020
Gender, recipient, male	0.024	1.071	1.009	1.137
Recipient BMI, kg/m2	0.250	1.003	0.998	1.007
Recipient Race/Ethnicity	0.346	1.008	0.992	1.024
Etiology (recipient)	0.000	1.017	1.011	1.023
MELD	0.000	1.011	1.005	1.018
Era	0.000	0.948	0.942	0.955
Donor age, years	0.000	1.009	1.007	1.011
Gender, donor, male	0.980	0.999	0.942	1.060
Donor BMI, kg/m2	0.166	1.004	0.998	1.009
Donor Race/Ethnicity	0.001	0.974	0.958	0.990
CIT	0.585	1.002	0.994	1.012
DCD graft	0.000	1.556	1.299	1.863

^1^Multivariate Cox regression model.

DCD, Donation after circulatory death; DBD, donation after brainstem death; LT, liver transplantation; BMI, body mass index, MELD, Model For End-Stage Liver Disease; CI, confidence interval; HR, hazard ratio.

### Identification of protective factors

We compared characteristics of DCD-LTs in MELD≥35 versus MELD<35 patients in the UNOS cohort (n=5,790 DCD-LTs) (**Supplementary Table 5**). The MELD≥35 DCD-LT group included younger donor age, lower donor BMI, and lower recipient age. More retransplants, longer donor hepatectomy times, longer CITs, and more patients on life support at the time of transplant were also observed in the latter group. **Supplementary Figure 3A** shows patient survival for the two groups with early mortality observed in the MELD≥35 group. We compared DCD-risk scores [as described in our previous publication (3)] between MELD≥35 and MELD<35 recipients. UK-, Total-WIT-, and UC-DCD scores were higher in MELD≥35 DCD-LTs, indicating higher risk pattern. To identify potential protective factors for non-death censored graft survival, we analyzed variables known to influence DCD outcomes in the MELD≥35 cohort. We found that using a donor of less than 30yo for a DCD-LT with MELD≥35 independently reduced the risk of graft loss by 30% (HR, 95%CI: 0.7 (0.9-0.5), p=0.019) (**Table 7**). Retransplant status was associated with a more than doubled risk of adverse event (HR, 95%CI: 2.1 (1.4-3.3), p=0.001).

**Table 7:** Estimated hazard ratios for liver graft survival using a uni- and multivariate Cox proportional hazard model in MELD≥35 recipients in the UNOS cohort (n = 375).

	p-value	Univar. HR^1^	95.0% CI		p-value	Multivar. HR^2^	95.0% CI	
			Lower	Higher			Lower	Higher
Recipient age at transplant, years	0.737	1.002	0.989	1.016				
Retransplant	0.001	2.102	1.361	3.245	0.001	2.144	1.388	3.31
Recipient BMI, kg/m2	0.458	0.991	0.966	1.016				
HBV core+	0.263	1.764	0.653	4.764				
Recipient life support	0.177	1.025	0.989	1.062				
Recipient underlying disease								
- Low risk - Standard risk - High risk	1 (Ref.) 0.141 0.205	1.291 0.279	0.919 0.039	1.812 2.006				
Donor age, <30years	0.024	0.704	0.955	0.518	0.019	0.693	0.940	0.510
Donor BMI, kg/m2	0.112	1.021	0.995	1.047				
Functional warm ischemia time	0.752	1.003	0.986	1.019				
Donor hepatectomy time	0.732	0.998	0.988	1.009				
TIPS	0.407	1.038	0.951	1.133				
CIT	0.446	0.973	0.907	1.044				

^1^Uni- and ^2^Multi-variate Cox regression model.

DCD, Donation after circulatory death; LT, liver transplantation; BMI, body mass index, MELD, Model For End-Stage Liver Disease; HR, hazard ratio.

## Discussion

DCD liver grafts are rarely used in high MELD recipients, presumably due to concern over the potential of more significant reperfusion syndrome’s effect on patient and graft survival, and renal and biliary complications (2, 5, 13–16). Recipients with a high MELD are almost uniformly bypassed on match runs, and these livers are preferentially transplanted into recipients with a lower MELD. This corresponds to the clinical notion that a sicker patient is less able to tolerate the complications associated with a marginal graft. It is, however, possible that highly selected DCD liver grafts could offer equivalent outcomes to DBD grafts in high MELD recipients. We, therefore, aimed to determine if DCD-LTs are feasible in high MELD recipients and measure the morbidity/mortality of such an approach with a focus on postoperative consequences of ischemia/reperfusion, renal function, and biliary complications. Here we show that cold storage DCD-LT can be safe in selected patients with a high MELD with an acceptable impact on renal recovery and biliary complications. In the absence of selection, DCD-LTs outcomes remain worse than those of DBD-LTs for high MELD patients.

Organ allocation policies favor the sickest patients, i.e, the highest MELD patients. As a consequence, transplant surgeons and physicians may feel that higher-quality organs will become available in a timely fashion. This may be supported by offer acceptance/decline patterns that have been observed after allocation policy changes. The literature to guide the utilization of DCD grafts in high MELD recipients is limited. In the U.S., between 2005 to 2021, DCD-LT for MELD≥35 represented only 0.4% of deceased donor liver transplants. Recipients with a high MELD have been shown to benefit the most from liver transplants (17), and the survival benefit of undergoing a DCD liver transplant compared to remaining on the waiting list increases with MELD (18). The rationale for not using a DCD graft for high MELD recipients is that they will get acceptable DBD offers. For a candidate with a MELD ≥ 30, the probability of getting a DBD-LT by one year is 68%, and the likelihood of dying on the waiting list is 14% (18). There is a ~3% survival benefit of accepting a DCD graft for patients, which increases to ~10% for those with a MELD≥35 (18). MELD is not the only independent predictive factor for DCD-LT graft loss (3) and some DCD scores include MELD in their algorithm (2, 4) and some do not (3, 5). Our results suggest that highly selected DCD liver grafts can potentially be used in high MELD recipients, bringing nuances to the general principle arguing against this practice. The rationale for using DCD grafts is, therefore, greater in regions with high median MELD at transplant, where some patients have no other alternative. This question will become of even greater importance with the rapidly increasing use of machine perfusion (7). The paucity of data to guide clinical decisions and the evidence showing comparable outcomes between DCD- and DBD-LTs (19) constitute a valid rationale to explore the possibility of utilizing a DCD graft in high MELD recipients.

We found equivalent one- and five-year survival between DCD and DBD-LT recipients with MELD≥35 in the UCSF cohort. The difference between the two groups was small, 90% vs. 91% at one-year (90 vs. 95% after adjustment). The need for long-term renal replacement therapy did not differ between DCD-LT recipients and DBD-LT recipients. Generally, our strategy has been to restrict criteria for DCD liver acceptance to donor age <40 with minimally fatty grafts for recipients with MELD≥35. A comparison between the baseline characteristics of all DCD- and DBD-LTs with MELD≥35 showed that DCD-LTs had a lower mean MELD score. However, even in the propensity-matched cohorts and in the multivariate model analysis, DCD graft type was not associated with patient survival. Independent factors associated with patient survival were as expected, namely: younger donor/recipient’s age, shorter CIT, and recent transplant era.

Our analysis of the UNOS population confirmed that the use of DCD grafts in recipients with MELD≥35 was rare. We also confirmed that DCD-LTs were from younger donors with shorter CIT. The multivariate analysis in the UNOS cohort confirmed the predictive factors for liver recipient survival from the UCSF cohort with the exception of CIT, which was no longer significant [we attribute this to a known difference for CIT in DCD-LTs in our cohort versus the UNOS cohort (3)]. Also, DCD grafts were associated with lower survival in the UNOS cohort and not in the UCSF cohort, coinciding with our previous observations (3). For instance, MELD at transplant was higher in the UNOS cohort, and it may have therefore amplified the effect of the graft type as suggested by our sensitivity analysis. Additional predictive factors in the UNOS cohort (not present in the UCSF cohort) were MELD, cause of liver disease, donor ethnicity/race, and recipient’s gender. With regard to the latter differences, it is possible that we could not observe those in our limited cohort due to smaller numbers. Donor and recipient selection and assessment seem key to ensuring optimal outcomes in high MELD recipients. An analysis of MELD≥35 recipients in the UNOS cohort allowed us to identify that a donor of less than 30yo independently reduced the risk of graft loss by 30%. On the other hand, retransplant status was associated with a more than doubled risk. Interestingly, it appears that in the MELD≥35 DCD-LT group more retransplants, more patients on life support at the time of transplant, longer CIT and longer donor hepatectomy times were tolerated compared to recipient with MELD<35. This could be the result of the scarcity of deceased donor allografts, pushing teams to accept DCDs for sick patients in the absence of any other option. Of note, the initial ischemia-reperfusion observed in DCD-LTs did not seem to have an impact in the long run, given the similar rejection rates between the two groups.

Our retrospective single-center study design introduces inherent limitations. In addition, the modest number of recipients in the UCSF cohort can contribute to type II statistical error. Nevertheless, variations in survival between groups were small and did not vary after adjustments. Protracted renal dysfunction was rare in this cohort, but similar to DCD-LT in low MELD recipients, more research is needed to help understand the specific characteristics that might portend worse renal function after transplant after DCD-LT in high MELD recipients. Determining the precise factors that explained the difference between the UCSF and UNOS cohorts remained difficult. Besides lower donor/recipient age and lower donor BMI, the level of granularity in both cohorts did not allow to identify more refined factors. Also, the influence of intra- and post-operative factors will need further study to be determined. The analysis of MELD≥35 versus MELD<35 in the UNOS cohort allowed to identify much lower donor/recipient age and lower donor BMI as potential protective factors. A long study period was required to capture enough cases, and this can represent a limitation as well, given the constant improvement in techniques and outcomes. To address this, we performed a sensitivity analysis to assess differences in predictors of good outcomes in MELD≥35 patients transplanted in the period before and after 2012 and found no difference. Another discussion point is the potential risk of increased early/intraoperative death in DCD-LTs, e.g., we had two in our cohort is clear that very early DCD-LT mortality is increased in the UNOS cohort as well. Our perspective is that this risk will remain and that, given our high MELD waitlist mortality, the decision to proceed or not with the only graft available (i.e., a DCD graft) remains difficult. In this context, it is important to note that Taylor et al. showed that patients who received DCD livers have a lower risk of death than those who remained on the waiting list for a potential DBD organ (20). Hopefully, DCD-associated intra/peri-operative mortality risk will be reduced with the use of machine perfusion. Bile duct complications remain more frequent after DCDs, and this will also be in part corrected by machine perfusion (7, 8, 21). We previously demonstrated that half of these patients become stent free, although the remainder will either require long-term stents, retransplant, or will die from biliary complications (3). Lastly, our study precedes the 2020 allocation changes, and, therefore, our observations and conclusions could differ when applied to the current system. Since the allocation changes, we have seen only a marginal increase in cold ischemia time, that is not likely to influence our conclusions. However, timely access to liver offers for high MELD patients remains challenging.

In conclusion, in the absence of selection, DCD-LTs outcomes remain worse than those of DBD-LTs. DCD-LT can achieve excellent outcomes in recipients with MELD≥35 using strict selection criteria and static cold storage. In the UCSF cohort, length of stay, dialysis requirement after transplant, and patient and graft survival were equivalent between DCD- and DBD-LTs. Biliary complications occurred at the expected rate. This suggests high-quality DCD liver donors can be considered a viable transplant option for recipients with MELD≥35. This has implications for organ acceptance practices and the ongoing discussion about implementation of machine preservation (8).

## Data availability statement

The raw data supporting the conclusions of this article will be made available by the authors, without undue reservation.

## Ethics statement

The studies involving humans were approved by Institutional Review Board of the University of California, San Francisco. The studies were conducted in accordance with the local legislation and institutional requirements. The ethics committee/institutional review board waived the requirement of written informed consent for participation from the participants or the participants’ legal guardians/next of kin because of the retrospective nature of the data review.

## Author contributions

RM and GR designed the study. RM, MN, SS, SF, MT, CF, JR, NA, RH, and GR collected the data. RM analyzed the data. RM performed the statistical analysis. RM, MN, SS, SF, MT, CF, JR, NA, RH, and GR interpreted the data and wrote the manuscript. RM and GR have full access to all of the data in the study and take responsibility for the integrity of the data and the accuracy of the data analysis. All authors contributed to the article and approved the submitted version.

## Supplementary material

Supplementary Figure 1 Waitlist mortality and drop out for candidates with MELD≥35 in the UCSF cohort

Supplementary Figure 2
**(A, B)** Kaplan-Meier curves showing patient’s survival, stratified by 90-day creatinine levels. **(C, D)** Kaplan-Meier curves showing graft’s survival, stratified by type of graft.

Supplementary Figure 3
**(A)** Kaplan-Meier curves showing patient’s survival, stratified by DCD-LT with MELD≥35 versus MELD<35, and **(B)** after excluding DCD recipients who died or lost their graft <1y. DCD scores in MELD≥35 versus MELD<35.

Supplementary Figure 4 Kaplan-Meier curves showing patient’s survival stratified by the different eras, in **(A)** DCDs and DBDs.
