# Supplementary material for: DCD liver transplant in patients with a MELD over 35
Source: Front Immunol. 2023 Sep 4;14:1246867. doi: 10.3389/fimmu.2023.1246867 (PMC10507358; doi:10.3389/fimmu.2023.1246867)

Supplementary Table 1: Etiology of liver disease in the UCSF cohort.

	DCD (n=41)	DBD (n=1,767)
Unknown	0	466
	0.0%	26.4%
A1at	0	8
	0.0%	0.5%
AIH	4	63
	9.8%	3.6%
Biliary atresia	0	1
	0.0%	0.1%
Cholangiocarcinoma	0	2
	0.0%	0.1%
Cryptogenic	1	140
	2.4%	7.9%
Etoh	14	239
	34.1%	13.5%
HBV	1	162
	2.4%	9.2%
HCC	2	16
	4.9%	0.9%
HCV	14	418
	34.1%	23.7%
NASH	4	56
	9.8%	3.2%
PBC	0	89
	0.0%	5.0%
PSC	1	91
	2.4%	5.1%
Wilson	0	16
	0.0%	0.9%
Total	41	1767
	100.0%	100.0%

Global $p<0.001$

Supplementary Table 2: Etiology of liver disease in the UNOS cohort.

	DCD (n=375)	DBD (n=17,677)
Unknown	0	17
	0.0%	0.1%
A1AT	3	260
	0.8%	1.5%
Acute	41	423
	10.9%	2.4%
AIH	0	611
	0.0%	3.5%
AUTO	20	0
	5.3%	0.0%
Biliary atresia	3	46
	0.8%	0.3%
Crypt	16	807
	4.3%	4.6%
EtOH	109	4707
	29.1%	26.6%
HAV	0	44
	0.0%	0.2%
HBV	8	684
	2.1%	3.9%
HCC	31	1142
	8.3%	6.5%
HCV	55	3031
	14.7%	17.1%
HCV_H	1	58
	0.3%	0.3%
HDV	0	7
	0.0%	0.0%
Hemoc.	4	82
	1.1%	0.5%
NASH	27	1637
	7.2%	9.3%
Other	31	2743
	8.3%	15.5%
PBC	10	531
	2.7%	3.0%
PSC	11	663
	2.9%	3.8%
Tumor	1	20
	0.3%	0.1%
Wilson	4	164
	1.1%	0.9%
Total	375	17677
	100.0%	100.0%

Global p<0.001

Supplementary Table 3: Cause of death in the UCSF cohort

	DCD (n=41)	DBD (n=41)
Intraoperative death	2 (4.9)	0 (0.0)
Cardiac arrest	0 (0.0)	2 (4.9)
Respiratory failure	1 (2.4)	0 (0.0)
Biliary obstruction	1 (2.4)	1 (2.4)
Sepsis	0 (0.0)	1 (2.4)
Cancer	1 (2.4)	1 (2.4)
Intracerebral / stroke	1 (2.4)	0 (0.0)
Unknown	2 (4.9)	0 (0.0)

Supplementary Table 4: Estimated hazard ratios for liver recipient survival using a multivariate Cox proportional hazard model in the UNOS cohort, MELD 35-36 recipients only (n = 4,153).

	p-value	HR[1]	95.0% CI Lower	95.0% CI Higher
Recipient age at transplant, years	0.000	1.016	1.010	1.022
Gender, recipient, male	0.095	1.118	0.981	1.275
Recipient BMI, kg/m2	0.952	1.000	0.990	1.011
Recipient Race/Ethnicity	0.429	1.015	0.979	1.052
Etiology (recipient)	0.017	1.016	1.003	1.030
MELD	0.596	0.967	0.853	1.096
Era	0.000	0.933	0.919	0.946
Donor age, years	0.005	1.006	1.002	1.010
Gender, donor, male	0.713	0.976	0.857	1.111
Donor BMI, kg/m2	0.998	1.000	0.989	1.012
Donor Race/Ethnicity	0.130	0.972	0.938	1.008
CIT	0.603	1.005	0.986	1.025
DCD graft	0.098	1.401	0.939	2.091

[1]Multivariate Cox regression model.

DCD, Donation after cardiac death; DBD, donation after brainstem death; LT, liver transplantation; BMI, body mass index, MELD, Model For End-Stage Liver Disease; CI, confidence interval; HR, hazard ratio.

Supplementary Table 5: Characteristics of donation after cardiac death liver transplantation with MELD<35 versus ≥35 in the UNOS cohort.

Characteristics	DCD-LT with MELD≥35 (n = 375)	DCD-LT with MELD<35 (n = 5,415)	P-value[1]
UK-DCD score parameters			
Donor age	30.6 ± 11.8	35.6 ± 13.5	<0.001
Donor BMI, kg/m^2	25.6 ± 5.7	27.1 ± 6.1	<0.001
Functional donor warm ischemia time, min	18.4 ± 9.6	18.2 ± 7.2	0.701
Cold ischemia time, hours	6.3 ± 2.3	5.9 ± 2.0	0.004
Recipient age, years	51.2 ± 11.6	56.9 ± 9.3	<0.001
Retransplantation (%)<br>- No<br>- Yes	 341(90.9)<br>34 (9.1)	 5,351 (98.8)<br>64 (1.2)	<0.001
UCLA-DCD score and KCH-DCD score parameters			
Donor HBV core +<br>- No<br>- Yes	 369 (98.4)<br>6 (1.6)	 5,277 (97.5)<br>138 (2.5)	0.383
Donor hepatectomy time, minutes	37.3 ± 17.0	34.0 ± 15.2	0.001
Recipient BMI	28.8 ± 6.3	28.6 ± 5.5	0.553
Recipient underlying disease<br>- Low risk<br>- Standard risk<br>- High risk	 79 (21.1)<br>8 (2.1)<br>288 (76.8)	 1,227 (22.7)<br>34 (0.6)<br>4,154 (76.7)	0.003
Other parameters			
TIPS<br>- No<br>- Yes	 352 (93.9)<br>23 (6.1)	 4,969 (91.8)<br>446 (8.2)	0.170
Life support<br>- No<br>- Yes	 313 (83.5)<br>62 (16.5)	 5,354 (98.9)<br>61 (1.1)	<0.001

[1]Student t-test for continuous variables, X^2 test for binary or categorical variables (global p-value).

BMI, body mass index; DCD, donation after cardiac death; MELD, model for end-stage liver disease; HBV, hepatitis B virus; HCV, hepatitis C virus; Ref., reference; UK, United Kingdom; UCLA, University of California Los Angeles; KCH, King's College Hospital. Low, standard, and high risk according with KCH score.

Supplementary Figure 1

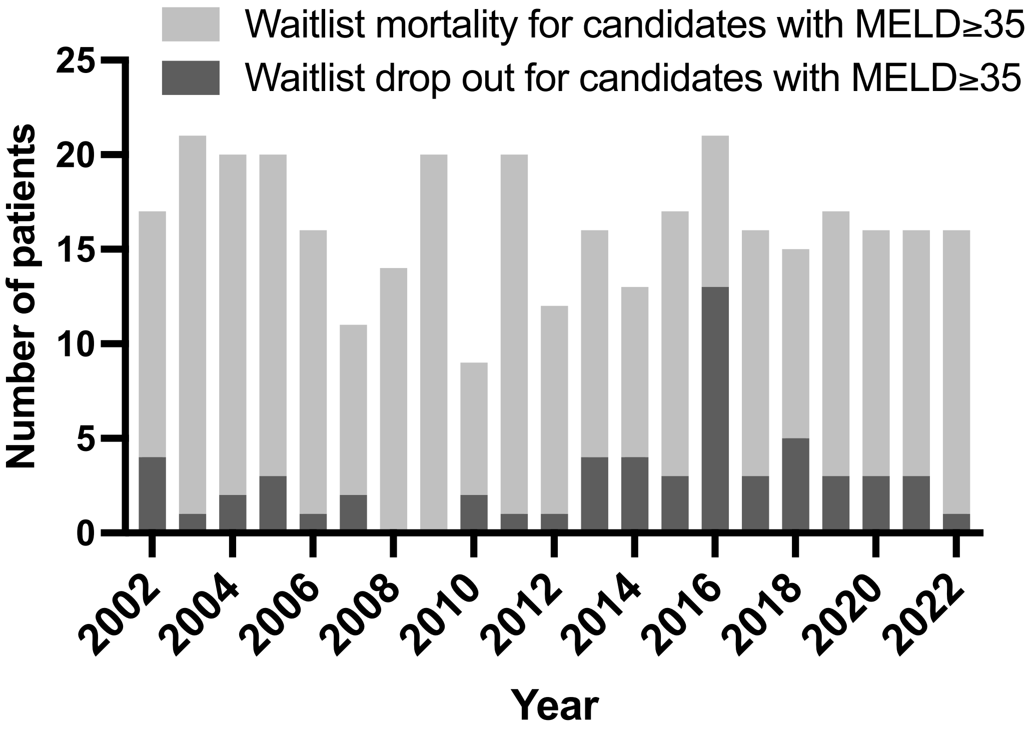

Supplementary Figure 2

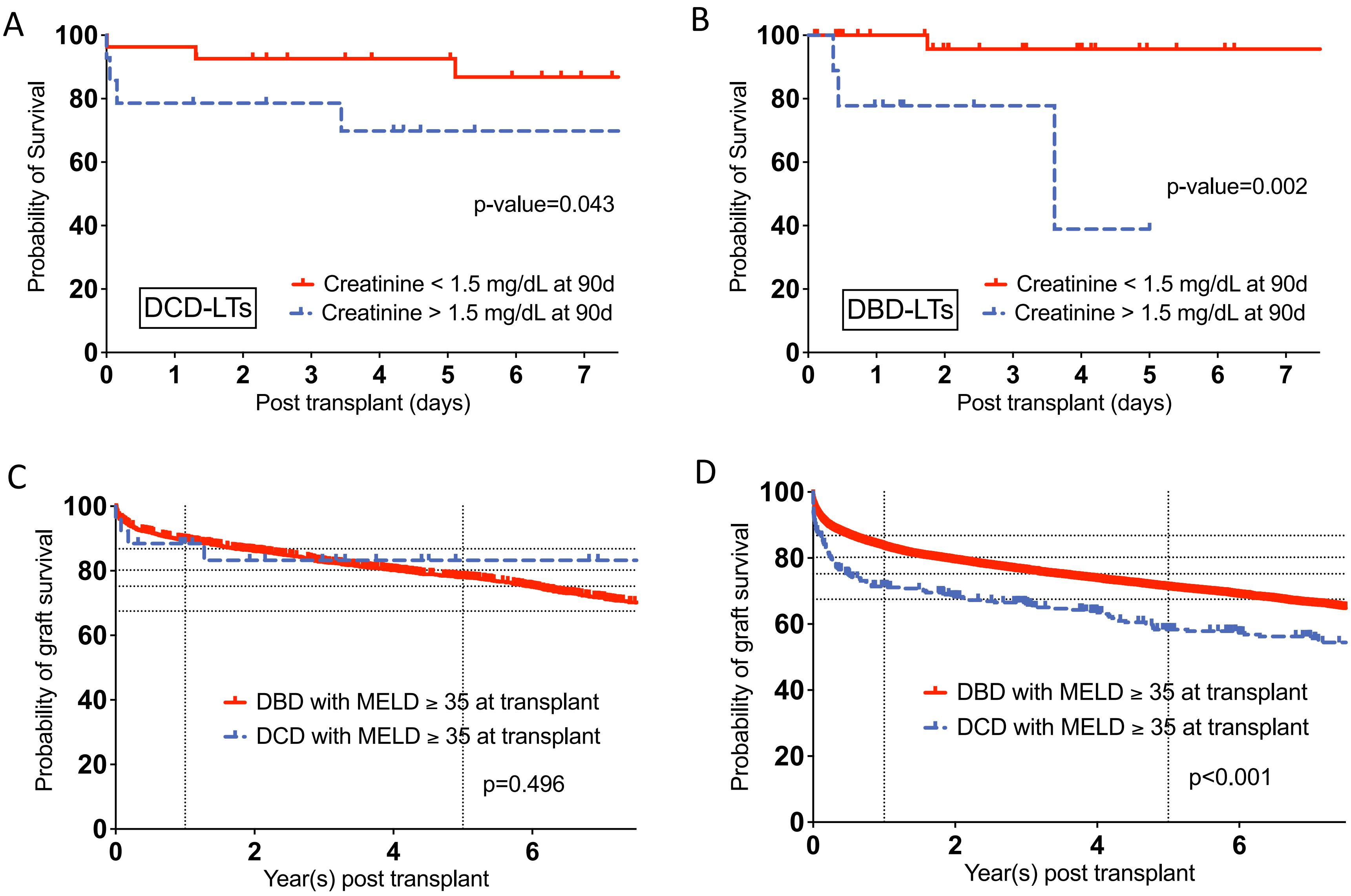

Supplementary Figure 3

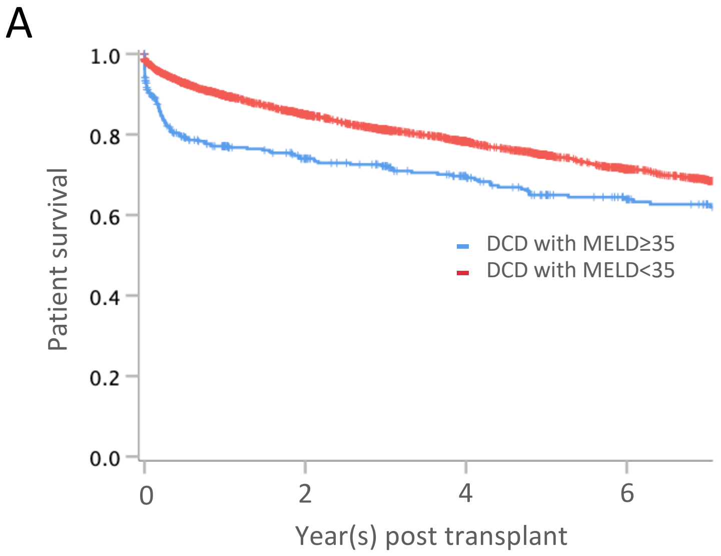

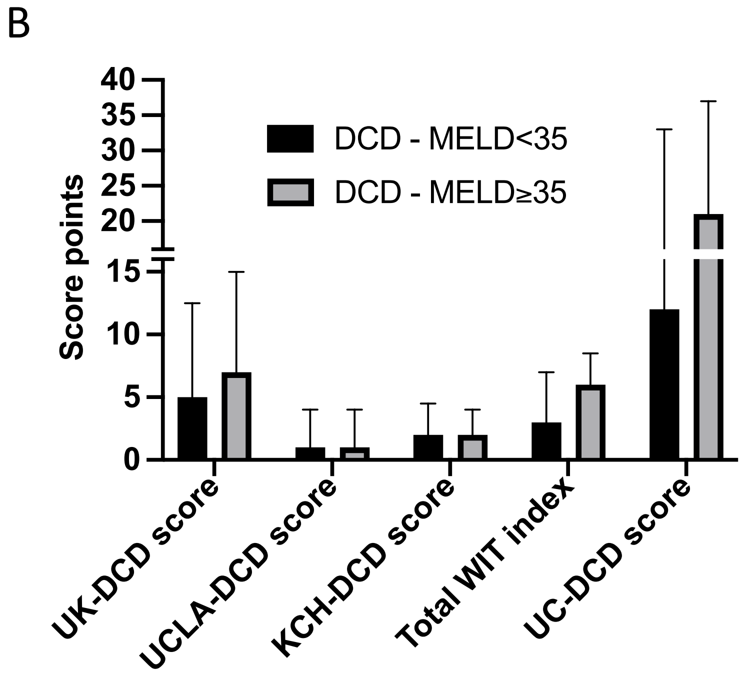

Supplementary Figure 4

A

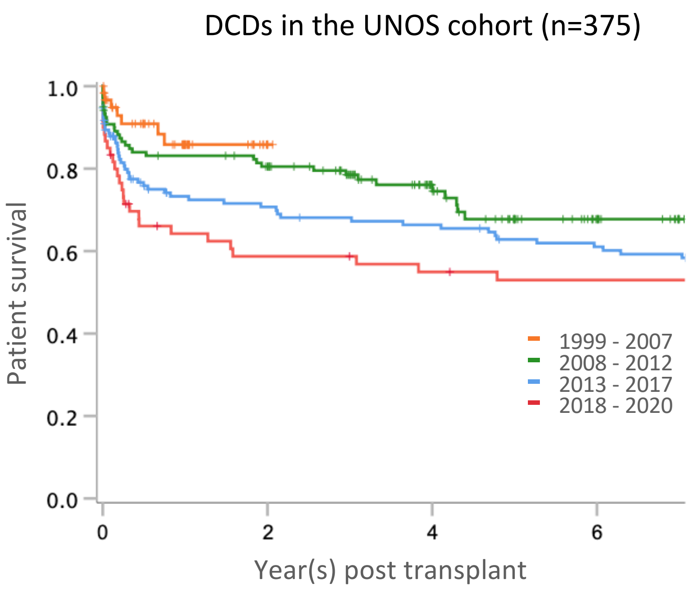

B

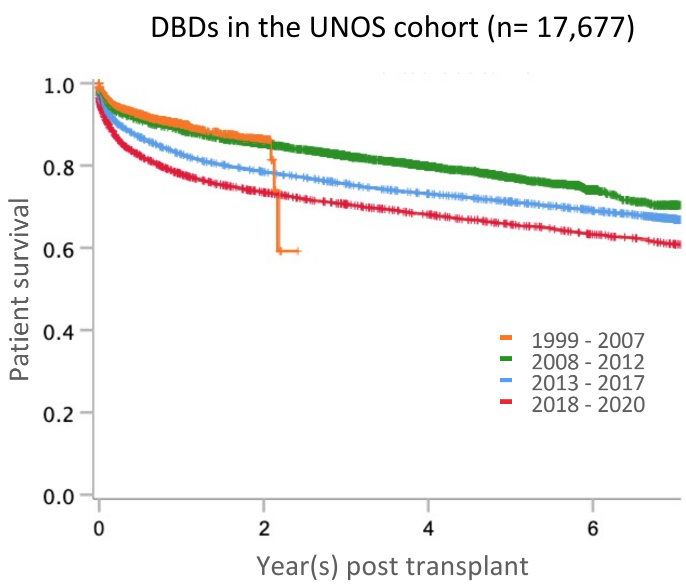

Supplement: Supplementary Figure 1 — Waitlist mortality and drop out for candidates with MELD≥35 in the UCSF cohort [file DataSheet_1.pdf]
